# Validation of a new kit for preeclampsia screening: A comprehensive analysis

**DOI:** 10.1016/j.heliyon.2024.e28080

**Published:** 2024-03-13

**Authors:** Min Zhu, Jumei Liu, Jiali Cao, Yan Ni, Mengqi Chang, Ruitong Chen, Zhiying Su, Weiwei Yu, Huiming Ye

**Affiliations:** a Department of Laboratory Medicine, Fujian Key Clinical Specialty of Laboratory Medicine, Women and Children's Hospital, School of Medicine, Xiamen University, Xiamen, China; b Department of Obstetrics, Women and Children's Hospital, School of Medicine, Xiamen University, Xiamen, China; c School of Public Health, Xiamen University, Xiamen, China

**Keywords:** Detection performance, Diagnostic performance, Preeclampsia, sFlt-1 and PlGF quantitative kits

## Abstract

**Objectives:**

Preeclampsia is a common pregnancy complication that significantly contributes to maternal mortality, perinatal mortality, and preterm delivery. The sFlt-1/PlGF (fms-like tyrosine kinase-1/placental growth factor) ratio has demonstrated robust diagnostic value for preeclampsia. This study assessed the analytical performance and diagnostic accuracy of a novel quantitative determination kit for sFlt-1 and PlGF for the diagnosis of preeclampsia.

**Methods:**

The detection performance of the test kit was validated using the Center for Medical Device Evaluation (CMDE) and Clinical and Laboratory Standards Institute (CLSI) documents. The test results were compared to those of the Elecsys immunoassay (Roche Diagnostics). Independent discovery and validation sets were used to analyze the diagnostic efficacy of the preeclampsia kit. The area under the curve (AUC) for preeclampsia at different gestational ages was calculated.

**Results:**

Correlation analysis between the test and Roche kits revealed a strong concordance (sFlt-1: r = 0.9966, P < 0.0001; PlGF: r = 0.9935, P < 0.0001). The AUCs for sFlt-1, PlGF, and the sFlt-1/PlGF ratio in diagnosing preeclampsia were 0.749, 0.795, and 0.834, respectively, in the discovery set and 0.729, 0.811, and 0.831, respectively, in the validation set. The corresponding results from the Roche kit were 0.741, 0.795, and 0.829, respectively, and 0.761, 0.864, and 0.844, respectively.

**Conclusions:**

Quantitative sFlt-1 and PlGF kits exhibited high levels of consistency with the Roche kits in terms of quantitative outcomes and diagnostic performance for preeclampsia.

## 1 Introduction

Preeclampsia is a prevalent pregnancy complication characterized by hypertension coupled with multi-organ involvement and damage. Its global incidence varies between 2% and 8% [[1], [2], [3]]. This condition remains a significant contributor to maternal and perinatal mortality and premature delivery. Patients with preeclampsia may exhibit elevated blood pressure, proteinuria, and symptoms such as headache, dizziness, nausea, vomiting, and upper abdominal discomfort after 20 weeks of gestation. The risk factors include age, multiple gestations, history of renal disease, diabetes, connective tissue disease, antiphospholipid antibody syndrome, chronic hypertension, and obesity. The precise etiology of preeclampsia remains unclear. However, abnormal development of the placental vasculature early in pregnancy may lead to relative placental hypoperfusion, hypoxia, and ischemia, consequently triggering the release of antiangiogenic factors into the maternal bloodstream. This imbalance between angiogenic and antiangiogenic factors significantly contributes to the onset and progression of preeclampsia [4].

Placental growth factor (PlGF), a proangiogenic factor, and soluble fms-like tyrosine kinase-1 (sFlt-1), an antiangiogenic factor, are produced by the placenta. sFlt1 is a splice variant of the vascular endothelial growth factor receptor Flt1 and a potent inhibitor of vascular endothelial growth factor [5]. The antiangiogenic factor sFlt-1 binds to PlGF, neutralizing its proangiogenic effect. During normal pregnancy, sFlt-1 levels increase after 30–32 weeks of gestation, whereas PlGF levels decline after 30 weeks [1]. In preeclampsia, syncytiotrophoblast dysfunction leads to the elevated expression and release of the antiangiogenic factor sFlt-1 into the maternal circulation. sFlt-1 functions as an antiangiogenic agent by binding to circulating free PlGF, causing a substantial reduction in blood PlGF concentration in preeclampsia compared to that in normal pregnancies [6,7]. Prior research has indicated heightened sFlt-1 levels and diminished PlGF levels in patients with preeclampsia, even prior to clinical symptom manifestation, making them vital indicators for screening, predicting, and diagnosing preeclampsia [4]. Evaluating the sFlt-1/PlGF ratio can provide valuable insights into the risk of hypertensive disorders during pregnancy. This ratio serves as a valuable tool for predicting and diagnosing conditions such as preeclampsia, enabling the implementation of appropriate preventive measures and treatments to safeguard the health of both mother and baby [8]. In a multicenter case-control study by Verlohren et al., distinct cutoffs for the sFlt-1/PlGF ratio were established for the early (20 + 0 to 33 + 6 weeks) and late gestational phases (34 + 0 weeks to delivery). Cutoffs of ≤33 and ≥ 85 yielded sensitivity/specificity rates of 95%/94% and 88%/99.5%, respectively, between 20 + 0 and 33 + 6 weeks. For the period after 34 + 0 weeks, cutoffs of ≤33 and ≥ 110 resulted in sensitivity/specificity rates of 89.6%/73.1% and 58.2%/95.5%, respectively [8]. Moreover, the sFlt-1/PlGF ratio can aid the short-term prediction of disease progression [3]. A prospective, multicenter, observational PROGNOSIS study found that an sFlt-1/PlGF ratio ≤38 had a negative predictive value (NPV) (no preeclampsia diagnosis) of 99.3% (95% confidence interval [CI], 97.9–99.9) in the subsequent week for pregnancy between 24 and 36 + 6 weeks. The NPV remained high at two (97.9%), three (95.7%), and four weeks (94.3%) after testing [3]. The cutoff level of ≤38 is widely accepted as a cost-effective indicator for ruling out preeclampsia in patients with suspicion of the disease [9].

The existing diagnostic kits for preeclampsia have several shortcomings. These include potential lack of specificity, leading to misdiagnosis or underdiagnosis; insufficient sensitivity for early signs of preeclampsia, resulting in delayed or inaccurate diagnosis; high cost, which limits widespread clinical adoption; complexity of operation, requiring specialized skills and training; difficulty in sample handling, such as strict requirements for serum or urine collection and storage conditions, which may restrict their practical clinical application; challenging result interpretation, necessitating complex explanations or integration with other clinical information for accurate diagnosis; and slow update cycles, as some kits may not be promptly updated or adapted to new clinical needs and findings as preeclampsia research advances. The Elecsys immunoassay for sFlt-1 and PlGF, developed by Roche Diagnostics (Penzberg, Germany) and carrying Conformité Europe (CE) certification as an in vitro medical device, is extensively utilized for quantifying sFlt-1 and PlGF [10]. The automated Roche Elecsys sFlt-1/PlGF immunoassay has been incorporated into the official guidelines of numerous countries for diagnosing and predicting preeclampsia [11].

This study assessed the performance of a quantitative determination kit designed to measure sFlt-1 and PlGF levels, including a comparative analysis with the Roche Diagnostics Elecsys immunoassay for these biomarkers. Furthermore, we evaluated the diagnostic efficacy of the kit in patients with preeclampsia.

## 2 Materials and methods

### 2.1 Study population

Blood samples were obtained from pregnant women undergoing prenatal examinations at Xiamen Maternal and Child Health Hospital. This study comprised independent discovery and validation sets with a case-control design. The discovery set encompassed 173 women (November 2019 to October 2020), where plasma or serum was collected at different gestational weeks for reference value comparison between the two kits, as well as for assessing their diagnostic accuracy in preeclampsia. The validation set included 166 women (February 2022 to March 2023) and was used to confirm the diagnostic accuracy of the two kits for preeclampsia. The study recruited women who were (1) pregnant, (2) adult, and (3) at risk of preeclampsia or who met the diagnostic criteria for preeclampsia. Pregnant women at high risk but not diagnosed with preeclampsia were classified into the control group. The diagnostic criteria included hypertension (systolic blood pressure ≥140 mmHg and diastolic blood pressure ≥90 mmHg after 20 weeks of gestation) accompanied by proteinuria (≥300 mg in a 24-h urine collection, urinary protein/creatinine ratio ≥0.3, or ≥1+ on dipstick testing), or in cases without proteinuria, involvement of one or more vital organs or systems (e.g., heart, lung, liver, and kidney), abnormal changes in blood, and digestive or nervous system or placental-fetal involvement, as per the Guidelines for the diagnosis and treatment of hypertensive disorders in pregnancy (2020). The exclusion criterion was incomplete clinical background information. We aimed to diagnose preeclampsia in populations at high risk of eclampsia. Therefore, when selecting the control group sample, we did not exclude individuals aged ≥35 years, those with abnormal body mass index (BMI) ≥28 kg/m^2^ or <18.5 kg/m^2^, a medical or family history of preeclampsia, adverse pregnancy outcomes, assisted reproduction, genetic factors related to hypertension, interpregnancy interval >6 years or <1 year, and comorbidities (gestational diabetes), and other high-risk factors. Healthy controls for normal pregnancies were matched to cases during recruitment based on race, maternal age, and gestational age. Normal pregnancy was defined as term delivery without documented hypertension, proteinuria, or growth restriction during the ongoing pregnancy. Serum or EDTA plasma samples were separated by centrifugation (800 g, 10 min) and stored at −20 °C following kit manufacturer instructions.

This study was approved by the Ethics Committee of Xiamen Maternal and Child Health Hospital (KY-2021-013-H01, 2019-02-004-H01, 2019-02-003-H01) and was performed in accordance with the Helsinki Declaration revised in 2013. Medical records were deidentified for all personally identifiable information.

### 2.2 Assays

Quantitative kits for sFlt-1 and PlGF were developed by Guangzhou DARUI Biotechnology Co., Ltd. Both reagents employ a double-antibody sandwich detection approach. sFlt-1 was detected using two mouse monoclonal antibodies, whereas PlGF was detected using a combination of goat polyclonal and mouse monoclonal antibodies. The Roche Elecsys immunoassay for sFlt-1 and PlGF served as the comparative kit. The chemiluminescence systems CARIS 200 (Xiamen UMIC Medical Instruments Co., Ltd., Xiamen, China) and Cobas E411 (Roche Diagnostics, Penzberg, Germany) were used. The reagents and calibration materials provided by each system were used according to the manufacturer's guidelines.

### 2.3 Performance validation

Several parameters were validated, including the calibration curve, limit of detection, reportable range, accuracy, precision, anti-interference capability, comparison between serum and plasma, and method comparison between the test and Roche kits. These validations were performed following the “guiding principles of performance analysis of diagnostic reagents in vitro” by the Center for Medical Device Evaluation (CMDE) in China and the Clinical and Laboratory Standards Institute (CLSI) documents.

**Calibration curve**: Calibration curves were generated using concentrations of calibration solutions (35, 180, 3300, 21000, and 100000 pg/mL for sFlt-1, and 20, 320, 1715, 14000, and 30000 pg/mL for PlGF). A four-parameter logistic curve was used for the fitting.

**Limit of detection:** The zero-concentration sample was used as the sample for detection; the measurement was repeated 20 times, and the mean (M), standard deviation (SD), and M +2 SD were calculated. According to the zero-concentration sample and the relative concentration-RLU value, results between adjacent calibrating products are subjected to two-point regression fitting to obtain a linear equation, and the RLU value of M+2SD is introduced into the above equation to obtain the corresponding concentration value, which is the blank limit. Five samples were prepared at low concentrations that approximated the detection limit, and each sample was tested five times. The test results are sorted in ascending order. If the number of test results below the blank limit of 6 pg/mL is ≤ 3, it indicates that the detection limit and its setting are reasonable. Five samples were prepared at concentrations approximating the limit of quantification, and each sample was tested five times. The number of measured values with a relative bias exceeding 20% should be ≤ 3. This indicated that the limit of the quantitation setting was reasonable.

**Reportable range:** A series of evaluation samples were prepared by selecting a high-value plasma sample (H) near the expected upper limit and a low-value plasma sample (L) near the expected lower limit. The samples were combined in various ratios as follows: 5 parts L, 4 parts L with 1 part H, 3 parts L with 2 parts H, 2 parts L with 3 parts H, 1 part L with 4 parts H, and 5 parts H. Each sample was subjected to triplicate measurements, and the mean was calculated. The RLU and matched concentrations were used as the y and x variables in a standard regression analysis to evaluate linearity. The lower limit of the reportable range was directly determined from the limit of detection. To extend the reportable range beyond the upper limit of the kit, 2 × dilutions were evaluated in serum samples at high levels. The reportable range was determined based on the percent coefficient of variation (CV) and relative bias within 15%.

**Accuracy:** We prepared high- and low-concentration calibration solutions and added 45 μL of the high-concentration calibration solution to 450 μL of the low-concentration calibration solution to obtain a mixed sample. We tested each sample three times and calculated their respective means. Recovery rates (R) between 85 and 115% were considered acceptable. $R=\frac{c\times \left(V_{0}+V\right)-c_{0}\times V_{0}}{V\times c_{s}}\times 100\%$ (*R*: recovery rate; *V*: volume of the high-concentration calibration solution; $V_{0}$: volume of the low-concentration calibration solution; *c*: measured concentration mean of the mixed sample; $c_{0}$: measured concentration mean of the low-concentration calibration solution; $c_{s}$: concentration of the high-concentration calibration solution.

**Precision**：The precision of the test kit was evaluated based on intra- and inter-day assays using high- and low-level samples. The samples were measured 20 times per day for the intra-assay. For the inter-assay study, the samples were analyzed on three consecutive days with five replicates.

**Anti-interference capability**: To determine whether increased concentrations of commonly occurring sample matrix components would interfere with the accuracy of the kit assay, the effects of elevated bilirubin, hemoglobin, triglyceride, rheumatoid factor antinuclear antibody, and human anti-mouse antibody levels were evaluated using additional interference. Anti-interference capability was analyzed by calculating the relative bias%.

**Plasma and serum**： Measurements of sFlt-1 and PlGF in EDTA plasma and serum were compared by testing 72 matched pairs of EDTA plasma and serum samples with dose values covering the entire reportable range of the assay, and the differences in the results were assayed. The logs of detection of sFlt-1 and PlGF were analyzed.

**Method comparison:** Methods from different manufacturers were compared. The sFlt-1 and PlGF levels were evaluated in parallel using the test and Roche kits in the present study, which included 173 patients, to analyze the correlation between the two kits. The log of sFlt-1 and PlGF measurements was used.

### 2.4 Statistical analysis

All statistical analyses were performed using SPSS software (version 22.0; SPSS Inc., Chicago, IL, USA) and GraphPad Prism 7 (GraphPad, La Jolla, CA, USA). P < 0.05 was considered statistically significant. Continuous variables are described using mean ± standard deviation (SD), and the independent sample *t*-test was used for continuous variables to compare patients and healthy controls. The Pearson contingency coefficient was used to determine the correlation between the test and Roche kits, and an equation was generated by simple linear regression analysis. Concurrence among assays was evaluated using the Bland-Altman plot analysis. Receiver operating characteristic (ROC) curves were constructed to calculate the area under the curve (AUC) and determine the best cutoff point, positive predictive value (PPV), negative predictive value(NPV), diagnostic accuracy, sensitivity, specificity, and likelihood ratio (LR) in the diagnosis of preeclampsia. We constructed ROC curves for three indicators, sFlt-1, PlGF, and sFlt-1/PlGF ratio, to assess their diagnostic effectiveness for preeclampsia. sFlt-1 utilized the original detection data, whereas PlGF was analyzed after taking the reciprocal for ROC curve analysis.

## 3 Results

### 3.1 Performance evaluation of the quantitative determination kit for sFlt-1 and PlGF

**Calibration curve**: Calibration curves of the analytes are shown in Supplementary fig. 1. Typical regression equations were derived as Y (sFlt-1)=(1.514 × 10e7-3000)/[1+(X/38155)^-1.183]+3000 (R square = 0.9999) and Y (PlGF) = *[(2.243 × 10e7-3000)/[1+ (X/42492)^-1.069]+3000 (R square = 0.9999) respectively.

**The limit of detection:** Twenty replicates of zero-concentration samples were used to determine the limit of the blank. The limits of the blank were 0.073 pg/mL for sFlt-1 and 0.001 pg/mL for PlGF (Supplementary table 1), which were lower than the limits of the blank in the Roche kit (6 pg/mL for sFlt-1 and 2 pg/mL for PlGF). Five low-level samples with approximate detection limits were tested, and each sample was tested five times to determine the limit of detection. The quantitative results of sFlt-1 were all <10 pg/mL, and the quantitative results of PlGF were all <3 pg/mL, respectively (Supplementary table 2). Five samples with approximate quantitation limits were tested, and each sample was tested five times to determine the limit of quantitation. The quantitative results of sFlt-1 were all <15 pg/mL, and the quantitative results of PlGF were all <10 pg/mL (Supplementary table 3).

**Reportable range:** Samples with different concentration levels, obtained by mixing high-level serum with low-level serum in different proportions, were used to validate the linear range of the test kit. These results are presented in Supplementary table 4 and Supplementary fig. 2. The typical regression equations of sFlt-1 and PlGF were Y = 0.9761X-15.57 (R^2^ = 0.9992) and Y = 0.9702X-20.06 (R^2^ = 0.995), respectively. The linear range of sFlt-1 and PlGF verified in this study was 1197.4–17677.89 pg/mL and 11.6–2422.3 pg/mL, respectively, which was in line with the linear change and passed the verification. The lower limit of the reportable range was directly determined from the limit of detection. The 4 × onboard dilution (bias% within 1/2TEa) extended the upper end of the reportable range of sFlt-1 to 70711.56 pg/mL. Similarly, the upper end of the reportable PlGF range was extended to 9689.2 pg/mL (Supplementary table 5).

**Accuracy:** The accuracy of the assay was evaluated using a recovery test. The mean recovery percentages were 99.83% and 105.40% for sFlt-1 and PlGF, respectively, indicating acceptable accuracy of the kit (Supplementary table 6).

**Precision:** The precision results are summarized in Supplementary table 7. The CV of intra- and inter-assay precision of sFlt-1 was 2.06%–4.08% and 3.63%–4.35% respectively. The CV of the intra- and inter-assay precision of PlGF was 2.32%–5.28% and 1.71%–3.76%, respectively.

**Anti-interference capability:** Analysis of interfering substances revealed that the bias% was within ±10% for both sFlt-1 and PlGF, indicating the absence of a statistically significant difference between serum samples without interferences and samples containing bilirubin at 817 μmol/L, hemoglobin at 18 g/L, triglyceride at 21.54 mmol/L, rheumatoid factor at 1500 U/mL, antinuclear antibody at 500 ng/mL, or human anti-mouse antibody at 500 ng/mL (Supplementary table 8).

**Plasma and serum:** The results (Supplementary fig. 3) indicated a good correlation between the measurements of EDTA plasma and serum samples (sFlt-1: r = 0.9936, P < 0.0001, PlGF: r = 0.9944, P < 0.0001), and the regression equation obtained was Y = 0.9949X+0.03074 (R square = 0.9871, P < 0.0001) for sFlt-1 and Y = 0.9934X+0.02678 (R square = 0.9942, P < 0.0001) for PlGF. The Bland-Altman analysis demonstrated that for sFlt-1, the mean bias ±SD between the EDTA plasma and serum samples was −0.01366 ± 0.03778 log10 pg/mL and that the limits of agreement were −0.08771 to 0.06038, and for PlGF, the mean bias ±SD was −0.01164 ± 0.04228 log10 pg/mL and the limits of agreement were −0.0945 to 0.07122, demonstrating satisfactory consistency between the measurement of EDTA plasma and serum samples.

**Fig. 1:**
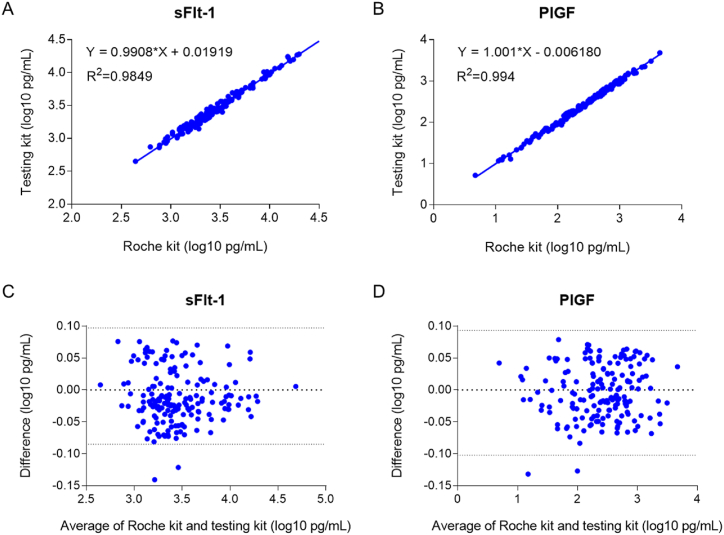
Method comparisons Linear regression analysis of the detection of sFlt-1 (A) and PlGF (B) between the test and Roche kits. Bland-Altman analysis for sFlt-1 (C) and PlGF (D) detected by the test and Roche kits.

**Method comparisons:** To compare the two methods, 173 serum samples were analyzed using the test and Roche kits. Satisfactory relevance and consistency were observed (Fig. 1). The correlation study with the test kit and Roche kit demonstrated similarity between the two methods (sFlt-1: r = 0.9966, P < 0.0001; PlGF: r = 0.9935, P < 0.0001). The regression equations of the logarithmically transformed results of the two methods were Y = 0.9908X+0.01919 (R^2^ = 0.9849, P < 0.0001) for sFlt-1(Fig. 1A), and Y = 1.001X-0.00618 (R^2^ = 0.994, P < 0.0001) for PlGF(Fig. 1B). The Bland-Altman plot showed the mean bias ±SD between two sFlt-1 quantitative kits was −0.01252 ± 0.04125 log10 pg/mL, and the limits of agreement were −0.09336 and 0.06832(Fig. 1C). The mean bias ±SD between two PlGF quantitative kits was −0.002635 ± 0.04183 log10 pg/mL, and the limits of agreement were −0.08461 and 0.07935(Fig. 1D).

The test and Roche kits provided the respective reference ranges of sFlt-1 and PlGF for different gestational weeks (5th to 95th percentile of normal pregnancies) according to the manufacturer's instructions. The test kit detected 56 abnormal samples (not within the reference range) for sFlt-1 and 57 abnormal samples for PlGF in 173 test samples, whereas the Roche kit detected 56 abnormal samples for sFlt-1 and 56 abnormal samples for PlGF. The results showed that the two kits had high positive, negative, and total coincidence rates for determining PLGF and sFlt-1 (Supplementary tables 9 and 10).

### 3.2 Diagnostic performance and cutoff values

**Fig. 2:**
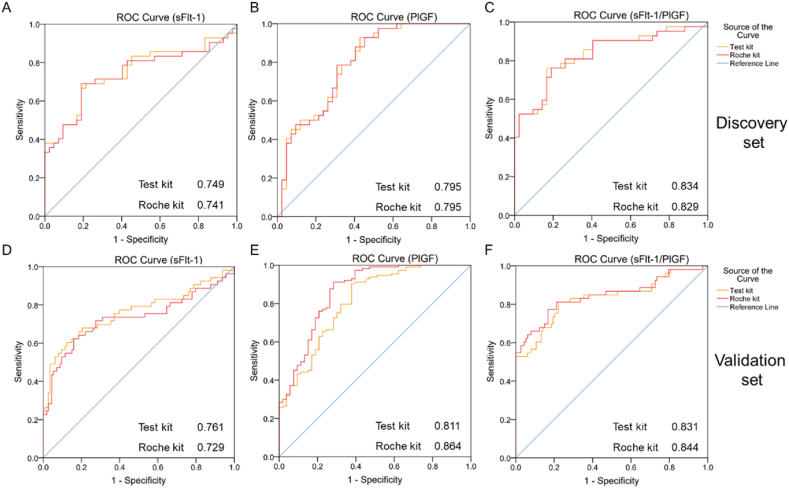
Receiver operator characteristic (ROC) curves of the three indices Area under the curve (AUC) of sFlt-1 (A), PlGF (B), and sFlt-1/PlGF ratio (C) in the discovery set, and sFlt-1 (D), PlGF (E), and sFlt-1/PlGF ratio (F) in the validation set.

The clinical characteristics of the women with preeclampsia and their matched controls are shown in Table 1. Among the 173 samples collected from the discovery set, 42 were diagnosed as preeclampsia. Table 2 The age of these pregnant women was 31.74 ± 5.61 years, and their gestational weeks were 34.76 ± 3.15 weeks. We selected 42 normal pregnant women in the control group to match the age and gestational weeks of the preeclampsia patients. The age was 31.88 ± 3.74 years, and the gestational age was 33.72 ± 3.84 weeks in the control group. All participants in the case and control groups were of Han nationality. There was no significant difference in maternal age (31.74 vs. 31.88 years, P = 0.891), gestational age (34.76 vs. 33.72 weeks, P = 0.180), or pre-gestation BMI (23.34 vs. 21.94 kg/m^2^, P = 0.121) between the case and control groups. To determine the diagnostic performance of the three indices (sFlt-1, PlGF, and sFlt-1/PlGF ratio) and establish cutoff values for use in a clinical setting, we constructed ROC curves using data from the 42 normal controls and 42 women with preeclampsia (Fig. 2, A-C). The AUCs of sFlt-1, PlGF, and the sFlt-1/PlGF ratio were 0.749, 0.795, and 0.834, respectively. Thus, the AUC of the sFlt-1/PlGF ratio was the highest of the three indices. The results were similar to those of the Roche kit, in which the AUC of sFlt-1, PlGF, and the sFlt-1/PlGF ratio were 0.741, 0.795, and 0.829, respectively. The sensitivity/specificity of the test kit for the diagnosis of preeclampsia at the cutoff values of sFlt-1, PlGF, and the sFlt-1/PlGF ratio were 69.0/78.6% (cutoff: >3844.71), 92.9/57.1% (cutoff: <157.48), and 76.2/83.3% (cutoff: >18.37), respectively. The sensitivity/specificity of the Roche kit for the diagnosis of preeclampsia at the cutoff values of sFlt-1, PlGF, and the sFlt-1/PlGF ratio were 69.0/81.0% (cutoff: >4143.50), 92.9/54.8% (cutoff: <140.05), and 76.2/81.0% (cutoff: >18.11), respectively. Table 3 shows the results related to LR, PPV, and NPV. We maintained false positives at a constant level and evaluated the sensitivity of the three detection metrics (Supplementary table 11). The performance of the sFlt-1/PlGF ratio in the diagnosis of preeclampsia was superior to that of sFlt-1 or PlGF alone (see Table 4).

**Table 1:** Basic clinical characteristics of pregnant women in the preeclampsia and control groups in the discovery set.

Characteristics	Preeclampsia (n = 42)	Control (n = 42)	P-value
Maternal age (years)	31.74 ± 5.61	31.88 ± 3.74	0.891
Gestational age (weeks)	34.76 ± 3.15	33.72 ± 3.84	0.180
Pre-gestation BMI	23.34 ± 4.47	21.94 ± 3.47	0.121

**Table 2:** Basic clinical characteristics of pregnant women in the preeclampsia and control groups in the validation set.

Characteristics	Preeclampsia (n = 53)	Control (n = 113)	P-value
Maternal age (years)	31.83 ± 4.82	31.17 ± 4.43	0.384
Gestational age (weeks)	36.41 ± 2.01	36.05 ± 1.26	0.232
Pre-gestation BMI	23.56 ± 4.25	21.18 ± 2.98	<0.01

**Table 3:** Diagnostic accuracy of sFlt, PlGF, and the sFlt/PlGF ratio for diagnosing preeclampsia in the discovery set.

Groups	Cutoff	Sensitivity (%)	Specificity (%)	LR+	LR-	PPV (%)	NPV (%)	AUC
sFlt-1 (Test kit)	3844.71	69 (55.1–83)	78.6 (66.2–91)	3.222	0.394	76.3	71.7	0.749 (0.64–0.857)
PlGF (Test kit)	157.48	57.1 (42.2–72.1)	92.9 (85.1–100)	8	0.462	88.9	68.4	0.795 (0.699–0.891)
sFlt-1/PlGF (Test kit)	18.37	76.2 (63.3–89.1)	83.3 (72.1–94.6)	4.571	0.286	82.1	77.8	0.834 (0.747–0.922)
sFlt-1 (Roche kit)	4143.5	69 (55.1–83)	81 (69.1–92.8)	3.625	0.382	78.4	72.3	0.741 (0.63–0.852)
PlGF (Roche kit)	140.05	69 (55.1–83)	78.6 (66.2–91)	3.222	0.394	76.3	71.7	0.795 (0.699–0.89)
sFlt-1/PlGF (Roche kit)	18.11	76.2 (63.3–89.1)	81 (69.1–92.8)	4	0.294	80	77.3	0.829 (0.739–0.919)

**Table 4:** Diagnostic accuracy of sFlt, PlGF, and the sFlt/PlGF ratio for diagnosing preeclampsia in the validation set.

Groups	Cutoff	Sensitivity (%)	Specificity (%)	LR+	LR-	PPV (%)	NPV (%)	AUC
sFlt-1 (Test kit)	5929.625	60.4 (47.2–73.5)	87.6 (81.5–93.7)	4.873	0.452	69.6	82.5	0.761 (0.671–0.851)
PlGF (Test kit)	0.006	62.3 (49.2–75.3)	90.3 (84.8–95.7)	6.396	0.418	75	83.6	0.811 (0.739–0.883)
sFlt-1/PlGF (Test kit)	16.845	81.1 (70.6–91.7)	77.9 (70.2–85.5)	3.667	0.242	63.2	89.8	0.831 (0.756–0.907)
sFlt-1 (Roche kit)	4399	62.3 (49.2–75.3)	84.1 (77.3–90.8)	3.909	0.449	64.7	82.6	0.729 (0.633–0.825)
PlGF (Roche kit)	0.008	71.7 (59.6–83.8)	91.2 (85.9–96.4)	8.102	0.31	79.2	87.3	0.864 (0.8–0.927)
sFlt-1/PlGF (Roche kit)	23.17	77.4 (66.1–88.6)	83.2 (76.3–90.1)	4.601	0.272	68.3	88.7	0.844 (0.77–0.919)

The validation set comprised 53 pregnant women diagnosed with preeclampsia and 113 controls. The clinical characteristics of the women with preeclampsia and their matched controls are shown in Table 2. There was no significant difference in maternal age (31.83 vs. 31.17 years, P = 0.384) or gestational age (36.41 vs. 36.05 weeks, P = 0.232) between the case and control groups. The pre-gestation BMI (23.56 vs. 21.18 kg/m^2^, P < 0.01) was higher in the preeclampsia group. All participants in the case and control groups were of Han nationality. Among the 166 pregnant women included in the validation set, we observed 27 cases of preterm birth, with 25 in the preeclampsia group and two in the control group. Other pregnancy complications in both groups included anemia and diabetes. The ROC curves for sFlt-1, PlGF, and sFlt-1/PlGF ratios are shown in Fig. 2, D-F. The AUCs of sFlt-1, PlGF, and the sFlt-1/PlGF ratio for the test kit were 0.761, 0.811, and 0.831, respectively. The corresponding AUCs for the Roche kit were 0.729, 0.864, and 0.844, respectively. The sensitivity/specificity of the test kit for the diagnosis of preeclampsia at the cutoff values of sFlt-1, PlGF, and the sFlt-1/PlGF ratio were 60.4/87.6% (cutoff: >5929.63), 90.3/62.3% (cutoff: <176.02), and 81.1/77.9% (cutoff: >16.84), respectively. The sensitivity/specificity of the Roche kit for the diagnosis of preeclampsia at the cutoff values of sFlt-1, PlGF, and the sFlt-1/PlGF ratio were 62.3/84.1% (cutoff: >4399.00), 91.2/71.7% (cutoff: <130.45), and 77.4/83.2% (cutoff: >23.17), respectively. Table 3 shows the results related to LR, PPV, and NPV. The results comparing sensitivity while holding false positives constant are presented in Supplementary table 12.

**Fig. 3:**
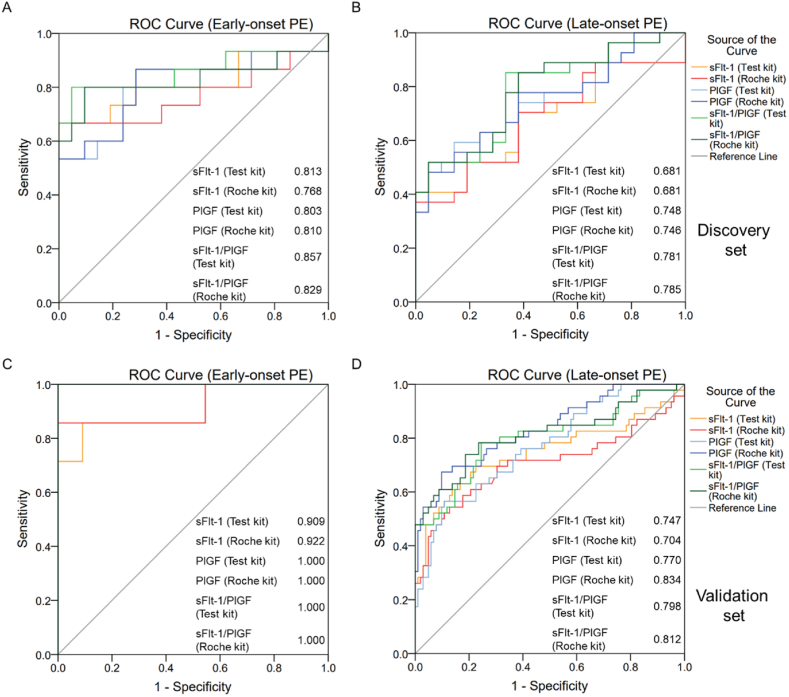
ROC curves in the diagnosis of preeclampsia at different gestational ages Area under the curve (AUC) of early-onset preeclampsia (A) and late-onset preeclampsia (B) in the discovery set, and AUC of early-onset preeclampsia (C) and late-onset preeclampsia (D) in the validation set.

Consistent with previous reports, the sFlt-1/PlGF ratio showed better diagnostic power for early-onset preeclampsia than for late-onset preeclampsia, and the AUC of the sFlt-1/PlGF ratio from the test kit was 0.857 for early onset (15 cases, 21 controls) and 0.781 for late-onset (27 cases, 21 controls) in the discovery set (Fig. 3A and B). In the validation set, the AUC of the sFlt-1/PlGF ratio from the test kit was 1.000 for early onset (cases: 7, controls: 11) and 0.798 for late onset (cases: 46, controls: 102) (Fig. 3, C-D).

## 4 Discussion

Preeclampsia remains an important cause of maternal and fetal/neonatal morbidity and mortality owing to the lack of effective treatments other than delivery. The pathogenesis of preeclampsia is multifactorial and not fully understood [12]. A previous study showed that serum concentrations of sFlt-1 increased, whereas PlGF concentrations decreased before the clinical symptoms of preeclampsia developed, resulting in an increased sFlt-1/PlGF ratio. An increased sFlt-1/PlGF ratio can predict subsequent preeclampsia [4]. Paolo et al. showed a correlation between an increased first-trimester preeclampsia risk and shorter pregnancy duration. Additionally, compared with the low-risk groups, the high-risk groups had significantly higher risks of spontaneous birth at various gestational weeks [13]. These findings underscore the clinical importance of the early risk assessment of preeclampsia in antepartum counseling and management.

In this study, the performance of a newly developed quantitative kit for sFlt-1 and PIGF from DARUI Biotechnology was evaluated, including the calibration curve, limit of detection, reportable range, accuracy, precision, anti-interference capability, and comparison of serum and plasma measurements. More importantly, the results of the test kits were compared to those of Roche kits. The Elecsys immunoassay for sFlt-1 and PlGF, developed by Roche Diagnostics, is the most widely used detection kit. The test kits were highly consistent with the control kits in terms of both quantitative results and the judgment of abnormalities at different pregnancy stages.

The cutoff value of the sFlt-1/PlGF ratio for predicting preeclampsia differs depending on the detection reagent used. Verlohren et al. established gestational phase-adapted cutoffs for the sFlt-1/PlGF ratio. The cutoffs at ≤33 and ≥ 85 resulted in the highest sensitivity/specificity in the early gestational phase (between 20 + 0 and 33 + 6 weeks), with a sensitivity/specificity of 95%/94% and 88%/99.5%, respectively, while the cutoffs at ≤33 and ≥ 110 after 34 + 0 weeks yielded a sensitivity/specificity of 89.6%/73.1% and 58.2%/95.5%, respectively [8]. In another study, the Elecsys immunoassay sFlt-1/PlGF ratio and Triage PlGF assay were compared. Similar to previous studies, the Elecsys immunoassay sFlt-1/PlGF ratio sensitivity/specificity was 94.0%/99.4% for early-onset preeclampsia and 89.5%/95.4% for late-onset preeclampsia [14]. In a study by Benton et al., the reassigned cutoff of 20 for Elecsys differed from the ratio cutoff of 85 defined in the product insert. The reassigned cutoff increased the sensitivity for any gestational age and early-onset and late-onset preeclampsia [15]. Nikuei et al. measured the levels of sFlt-1 and PlGF in 38 patients with preeclampsia and 20 normal-term pregnant women using an ELISA kit (R&D Systems). ROC curve analysis showed the diagnostic accuracy of the sFlt-1/PlGF ratio in preeclamptic patients, with an AUC of 0.90, the best cutoff value of 24.96, and sensitivity/specificity of 84.2%/85.0%. Kim et al. also used an ELISA kit from R&D Systems to measure the levels of sFlt-1 and PlGF. They found that the log[sFlt-1/PlGF] ratio was significantly higher in women with preeclampsia than in healthy controls. The ROC curve revealed a specificity of 78% with a sensitivity of 80.4%, and the best cutoff value for the log[sFlt-1/PlGF] ratio was 1.4 [16].

Our results are consistent with previous reports, which indicate that PlGF and sFlt-1 are altered in preeclamptic patients compared to those in normal pregnancies [4,17]. The best cutoff points for the sFlt-1/PlGF ratio of the Roche kit in this study were 18.11 and 23.17, which are close to the reassigned cutoff of 20 in the study by Benton et al. [15]. The cutoff points for the sFlt-1/PlGF ratio of the test kit were 18.37 and 16.84, and the corresponding sensitivity/specificity were 76.2/83.3% and 81.1/77.9%, respectively. Both assays had comparative discriminatory power in preeclampsia, as shown by their AUC results; however, the AUC, sensitivity, and specificity were not as high as those in previous studies. We believe that the reason is that the population included in this study did not exclude pregnant women with high-risk factors, such as age beyond 15–35 years, weight beyond 40–85 kg, history of abnormal pregnancy, medical conditions of pregnancy (including diabetes, hypothyroidism, and gestational cholestasis), poor delivery conditions (including pelvic abnormalities, multiple births, abnormal fetal position, and macrosomia), cicatricial uterus, excess or low amniotic fluid, in vitro fertilization or embryo transfer, abnormal reproductive structures, thalassemia, and placenta previa or hypofunction. The discriminatory power of early- and late-onset preeclampsia was analyzed. The AUC of sFlt-1/PlGF for early-onset preeclampsia was higher than that for late-onset preeclampsia (0.857 vs. 0.781, 1.000 vs. 0.798) using the test and Roche kits (0.829 vs. 0.785, 1.000 vs. 0. 812).

The limitations of this study include factors that could undermine the robustness and applicability of the findings. The small sample size was a limitation of this study. These preliminary data must be confirmed using a larger sample size to determine their validity. Moreover, high-risk factors were not excluded, potentially influencing the levels of the biomarkers sFlt-1 and PlGF, thus affecting diagnostic accuracy. Owing to the inclusion of high-risk pregnant women referred from primary care hospitals in the study sample, selection bias exists, which may limit the generalizability of the study results to the general pregnant population. Pregnant women with high-risk factors may differ in the occurrence and progression of preeclampsia, which could affect the generalizability of the study results. The case-control design employed may not fully mitigate confounding variables, and the study's reliance on specific assay kits limits its generalizability to other brands. The absence of long-term follow-up data further hinders our understanding of the development and prognosis of preeclampsia. While this study offers insights into sFlt-1 and PlGF assay kits for preeclampsia diagnosis, addressing these limitations through larger, more diverse samples and accounting for additional confounders is crucial for enhancing the reliability and applicability of future research findings.

In conclusion, we performed a transparent comparison between quantitative determination kits for sFlt-1 and PlGF developed by Guangzhou DARUI Biotechnology and the Elecsys immunoassay developed by Roche Diagnostics, as each assay's specific instructions were followed and performed on the same samples. The test kits were highly consistent with the control kit in terms of quantitative results and diagnosis of preeclampsia. The diagnostic performance was better for early-onset than for late-onset preeclampsia.

## Funding

This work was supported by the Major Science and Technology Project of the 10.13039/501100014125Fujian Provincial Health Commission, China (2021ZD01006, Huiming Ye founded by the Xiamen Municipial Health Commission, Huiming Ye), the Medical and Health Guidance Project of Xiamen, China (3502Z20214ZD1223, Jiali Cao), the Medical and Industrial Integration Guidance Project of Xiamen, China (3502Z20214ZD2143, Min Zhu), and the 10.13039/501100017686Fujian Provincial Health Technology Project, China (2019-2-52, founded by the Xiamen Municipal Health Commission, Jumei Liu).

## Data availability

The data associated with this study have not been deposited in any publicly available repositories. This information is available from the corresponding author upon request.

## Informed consent

Informed consent was obtained from all individuals included in this study.

## Ethical approval

This study was reviewed and approved by the Ethics Committee of the Xiamen Maternal and Child Health Hospital (approval number: KY-2021-013-H01, 2019-02-004-H01, 2019-02-003-H01).

## CRediT authorship contribution statement

**Min Zhu:** Investigation, Funding acquisition. **Jumei Liu:** Supervision, Funding acquisition. **Jiali Cao:** Writing – original draft, Funding acquisition. **Yan Ni:** Methodology, Formal analysis. **Mengqi Chang:** Data curation. **Ruitong Chen:** Data curation. **Zhiying Su:** Supervision, Resources. **Weiwei Yu:** Resources, Methodology, Conceptualization. **Huiming Ye:** Writing – review & editing, Funding acquisition, Conceptualization.

## Declaration of competing interest

The authors declare that they have no known competing financial interests or personal relationships that could have appeared to influence the work reported in this paper.
