# Supplementary material for: Validation of a new kit for preeclampsia screening: A comprehensive analysis
Source: Heliyon. 2024 Mar 13;10(6):e28080. doi: 10.1016/j.heliyon.2024.e28080 (PMC10963371; doi:10.1016/j.heliyon.2024.e28080)
Supplement: Multimedia component 1 [file mmc1.docx]

Validation of a New Kit for Preeclampsia Screening: A Comprehensive Analysis

Min Zhu^1*^, Jumei Liu^1*^, Jiali Cao^1*^, Yan Ni ^2*^, Mengqi Chang^3^, Ruitong Chen^3^, Zhiying Su ^2^, Weiwei Yu ^2#^, Huiming Ye ^1#^

^1^ Department of Laboratory Medicine, Fujian Key Clinical Specialty of Laboratory Medicine, Women and Children’s Hospital, School of Medicine, Xiamen University

^2^ Department of obstetrics, Women and Children’s Hospital, School of Medicine, Xiamen University

^3^ School of Public health, Xiamen University

^*^ Min Zhu, Jumei Liu, Jiali Cao and Yan Ni contributed equally to this work

^#^ Corresponding Authors: Huiming Ye Department of Clinical Laboratory, Women and Children's Hospital, School of Medicine, Xiamen University, Xiamen, 361003, China, Email: [yehuiming@xmu.edu.cn](mailto:yehuiming@xmu.edu.cn); Weiwei Yu Department of obstetrics, Women and Children’s Hospital, School of Medicine, Xiamen University, Xiamen, 361003, China, Email: weiwei1-3@163.com


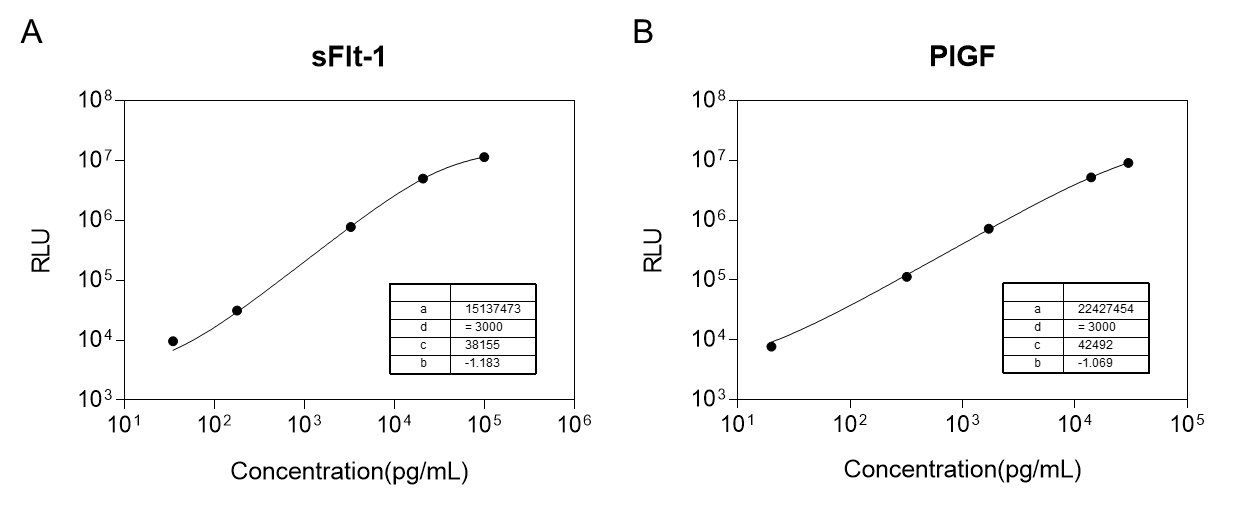


**Supplementary Figure 1** Calibration curve of analytes

Calibration curve of sFlt-1 (A) and PlGF (B)


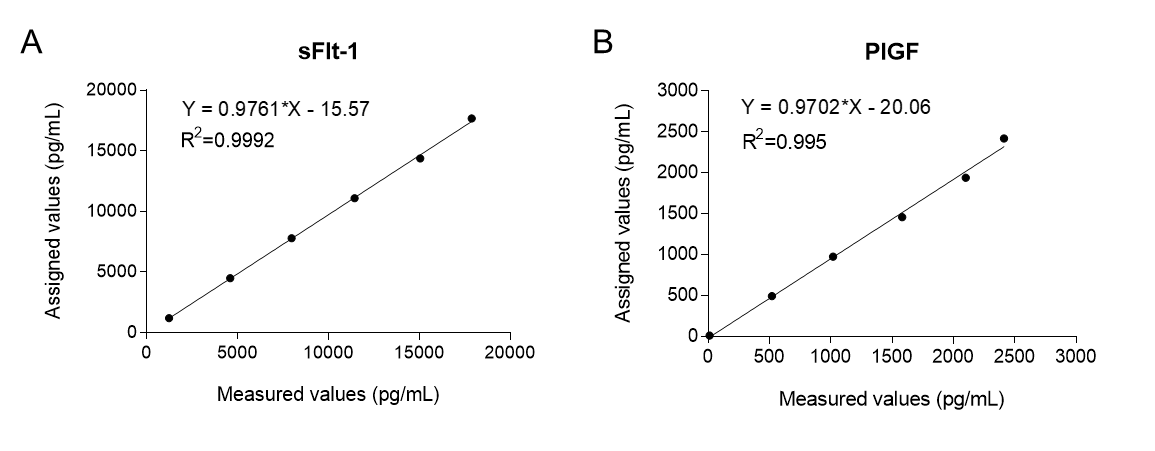


**Supplementary Figure 2** The linear range of the test kit

The linear range of sFlt-1 (A) and PlGF (B)


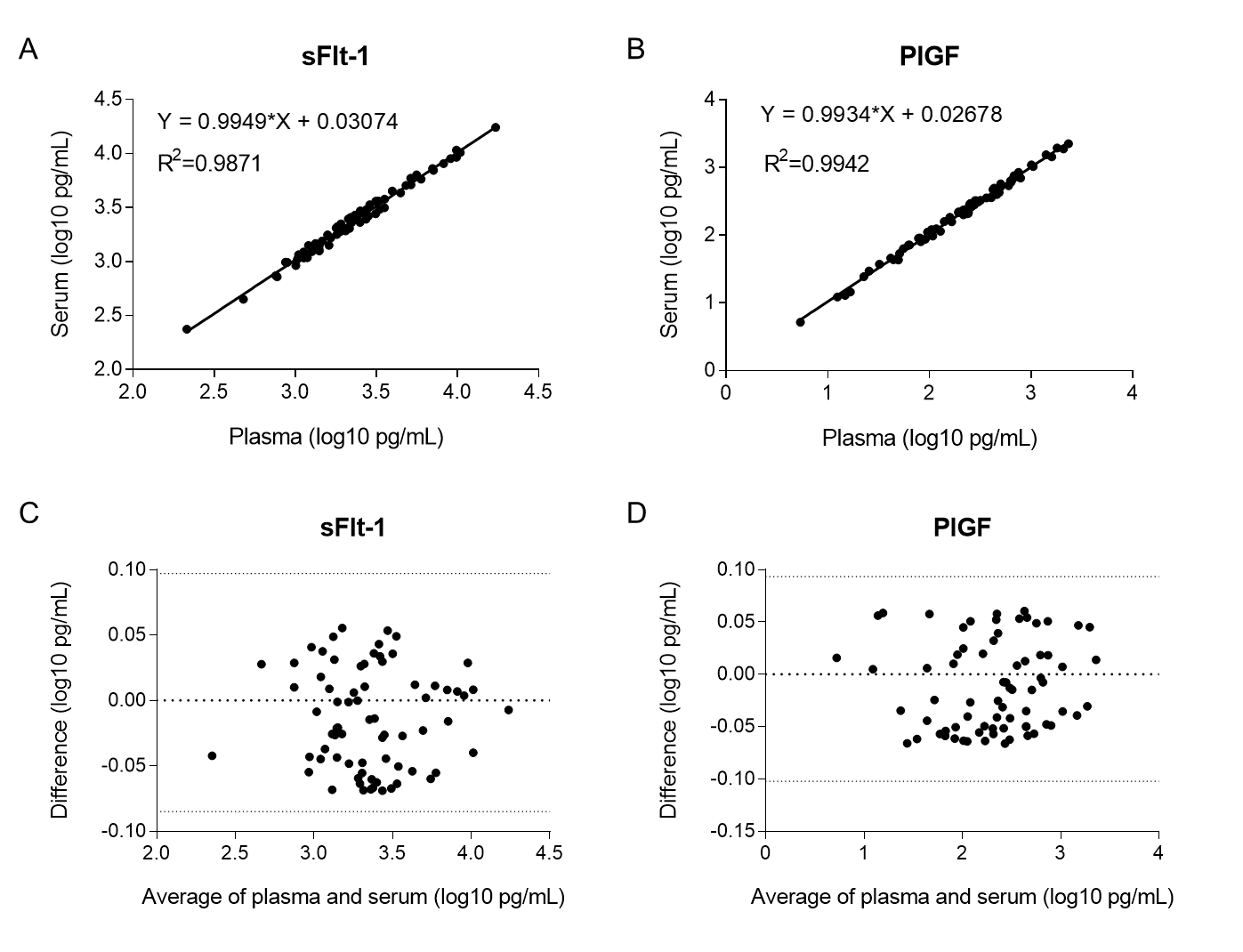


**Supplementary Figure 3** Comparation of plasma and serum

Linear regression analysis of the detection of sFlt-1 (A) and PlGF (B) in plasma and serum. Bland-Altman analysis for sFlt-1 (C) and PlGF (D) in plasma and serum.

**Supplementary Table 1** The limit of blank

|  | **Mean of RLU** | **SD** | **LOB (pg/mL)** |
| --- | --- | --- | --- |
| sFlt-1 | 263.4 | 33.23 | 0.073 |
| PlGF | 264.5 | 7.402 | 0.001 |

RLU, relative light units; SD, standard deviation; LOB, limit of blank.

**Supplementary Table 2** The limit of detection

|  | sFlt-1 (pg/mL) | PlGF (pg/mL) |
| --- | --- | --- |
| 1 | 9.92 | 2.68 |
| 2 | 9.86 | 2.67 |
| 3 | 9.83 | 2.67 |
| 4 | 9.73 | 2.65 |
| 5 | 9.67 | 2.62 |
| 6 | 9.45 | 2.6 |
| 7 | 8.91 | 2.6 |
| 8 | 8.69 | 2.59 |
| 9 | 8.66 | 2.56 |
| 10 | 8.58 | 2.55 |
| 11 | 8.53 | 2.53 |
| 12 | 8.42 | 2.52 |
| 13 | 8.41 | 2.51 |
| 14 | 8.25 | 2.51 |
| 15 | 8.22 | 2.46 |
| 16 | 8.07 | 2.44 |
| 17 | 7.99 | 2.43 |
| 18 | 7.97 | 2.42 |
| 19 | 7.89 | 2.39 |
| 20 | 7.62 | 2.38 |
| 21 | 7.34 | 2.38 |
| 22 | 7.24 | 2.38 |
| 23 | 7.1 | 2.37 |
| 24 | 7.1 | 2.35 |
| 25 | 7.07 | 2.33 |

**Supplementary Table 3** The limit of quantitation

| Sample | sFlt-1 | | PlGF | |
| --- | --- | --- | --- | --- |
|  | Measured value（pg/mL） | Bias % | Measured value（pg/mL） | Bias % |
| 1 | 13.80 | 5.34 | 4.98 | -0.40% |
|  | 11.75 | -10.25 | 5.10 | 2.00% |
|  | 13.04 | -0.47 | 4.56 | -8.80% |
|  | 12.23 | -6.63 | 4.52 | -9.60% |
|  | 12.40 | -5.31 | 4.76 | -4.80% |
| 2 | 12.52 | -4.40 | 4.91 | -1.80% |
|  | 13.90 | 6.09 | 5.09 | 1.80% |
|  | 14.82 | 13.16 | 5.25 | 5.00% |
|  | 13.54 | 3.41 | 4.92 | -1.60% |
|  | 14.39 | 9.84 | 5.03 | 0.60% |
| 3 | 13.91 | 6.24 | 5.49 | 9.80% |
|  | 12.72 | -2.89 | 4.63 | -7.40% |
|  | 13.51 | 3.17 | 5.47 | 9.40% |
|  | 13.20 | 0.79 | 4.93 | -1.40% |
|  | 11.23 | -14.26 | 5.29 | 5.80% |
| 4 | 13.32 | 1.70 | 5.04 | 0.80% |
|  | 13.35 | 1.91 | 4.61 | -7.80% |
|  | 12.49 | -4.61 | 5.34 | 6.80% |
|  | 11.60 | -11.44 | 5.33 | 6.60% |
|  | 12.94 | -1.19 | 5.26 | 5.20% |
| 5 | 11.55 | -11.78 | 4.64 | -7.20% |
|  | 14.11 | 7.75 | 4.78 | -4.40% |
|  | 14.45 | 10.31 | 4.82 | -3.60% |
|  | 14.55 | 11.07 | 5.23 | 4.60% |
|  | 12.11 | -7.56 | 5.13 | 2.60% |

**Supplementary Table 4** The linear range of the test kit

| **Dilution ratio**  **(Low : High)** | | **Assigned value（pg/mL）** | **Measured value（pg/mL）** | | | | **Relative deviation** |
| --- | --- | --- | --- | --- | --- | --- | --- |
|  |  |  | **1** | **2** | **3** | **Mean** |  |
| **sFlt-1** | 5：0 | 1197.40 | 1254.56 | 1214.97 | 1194.07 | 1221.2 | 1.99% |
|  | 4：1 | 4493.5 | 4609.91 | 4593.04 | 4545.30 | 4582.7 | 1.99% |
|  | 3：2 | 7789.6 | 8094.78 | 7910.48 | 7876.56 | 7960.6 | 2.20% |
|  | 2：3 | 11085.7 | 11611.21 | 11385.85 | 11292.84 | 11430.0 | 3.11% |
|  | 1：4 | 14381.8 | 15520.27 | 14652.90 | 14949.74 | 15041.0 | 4.58% |
|  | 0：5 | 17677.89 | 18010.65 | 18123.19 | 17477.89 | 17870.6 | 1.09% |
|  | **Regression equation: Y=0.9761X-15.57, R^2^=0.9992** | | | | | | |
| **PlGF** | 5：0 | 11.6 | 10.71 | 10.59 | 14.65 | 12.0 | 3.30% |
|  | 4：1 | 493.7 | 533.80 | 446.38 | 584.25 | 521.5 | 5.62% |
|  | 3：2 | 975.9 | 1025.88 | 1009.95 | 1022.71 | 1019.5 | 4.47% |
|  | 2：3 | 1458.0 | 1556.23 | 1507.69 | 1685.81 | 1583.2 | 8.59% |
|  | 1：4 | 1940.2 | 2064.32 | 2080.56 | 2159.83 | 2101.6 | 8.32% |
|  | 0：5 | 2422.3 | 2415.18 | 2432.73 | 2389.98 | 2412.6 | -0.40% |
|  | **Regression equation: Y=0.9702X-20.06, R^2^=0.995** | | | | | | |

**Supplementary Table 5** Reportable range

|  | | **Dilution ratio** | | | | | |
| --- | --- | --- | --- | --- | --- | --- | --- |
|  |  | 1/2 | 1/4 | 1/8 | 1/16 | 1/32 | 1/64 |
| **sFlt-1** | Mean±SD | 10615.79±317.8 | 6342.26±151.2 | 3718.86±184.8 | 2229.60±35.38 | 1350.01±27.07 | 801.17±10.67 |
|  | Restore  concentration | 21231.58 | 25369.03 | 29750.88 | 35673.60 | 43200.20 | 51275.11 |
|  | Assigned value | 22542.04 | 22542.04 | 22542.04 | 22542.04 | 22542.04 | 22542.04 |
|  | Bias | 5.81% | 12.54% | 31.98% | 58.25% | 91.64% | 127.46% |
|  | 1/2TEa | 15.00% | | | | | |
|  | Trusted maximum dilution | 1/4 | | | | | |
|  | Reportable range | 10.0~70711.56 pg/ml | | | | | |
| **PlGF** | Mean±SD | 1104.63±18.85 | 594.46±34.61 | 303.30±18.66 | 158.83±9.435 | 83.17±5.134 | 60.11±32.91 |
|  | Restore  concentration | 2209.26 | 2377.85 | 2426.40 | 2541.22 | 2661.30 | 3846.85 |
|  | Assigned value | 2100.02 | 2100.02 | 2100.02 | 2100.02 | 2100.02 | 2100.02 |
|  | Bias | 5.20% | 13.23% | 15.54% | 21.01% | 26.73% | 83.18% |
|  | 1/2TEa | 15.00% | | | | | |
|  | Trusted maximum dilution | 1/4 | | | | | |
|  | Reportable range | 3.0~9689.2 pg/ml | | | | | |

**Supplementary Table 6** Evaluation the accuracy of the assays by recovery test

|  | **Sample** | **Theoretical concentration (pg/mL)** | **Mean of measured concentration (pg/mL)** | **Recovery percentage** |
| --- | --- | --- | --- | --- |
| sFlt-1 | A | 20000.00 | 19997.13 | 99.83% |
|  | B | 10.00 | 9.96 |  |
|  | 50μL A+450μL B | 2009.00 | 2005.22 |  |
| PlGF | C | 5000 | 4911.98 | 105.40% |
|  | D | 10 | 10.39 |  |
|  | 50μL C+450μL D | 509 | 527.08 |  |

**Supplementary Table 7** Evaluation of Intra-assay­ and Inter-assay precision

|  | | **Intra-assay** | | | | | **Inter-assay** | | | | |
| --- | --- | --- | --- | --- | --- | --- | --- | --- | --- | --- | --- |
|  |  | Max  (pg/mL) | Min  (pg/mL) | Mean  (pg/mL) | SD | CV(%) | Max  (pg/mL) | Min  (pg/mL) | Mean  (pg/mL) | SD | CV(%) |
| **sFlt-1** | Low level | 1451.19 | 1326.58 | 1406.70 | 28.96 | 2.06 | 1508.74 | 1341.48 | 1421.42 | 61.79 | 4.35 |
|  | High level | 18921.77 | 15577.81 | 17309.75 | 706.5 | 4.08 | 18836.51 | 16683.41 | 17842.86 | 645.38 | 3.62 |
| **PlGF** | Low level | 17.32 | 14.47 | 15.71 | 0.83 | 5.28 | 21.89 | 19.28 | 20.77 | 0.78 | 3.76 |
|  | High level | 1057.97 | 966.75 | 1025.57 | 23.81 | 2.32 | 1239.50 | 1163.32 | 1201.90 | 20.60 | 1.71 |

**Supplementary Table 8** Evaluation of anti-interference capability

| **Interference** | | **sFlt-1** | | **PlGF** | |
| --- | --- | --- | --- | --- | --- |
|  |  | Sample 1 | Sample 2 | Sample 3 | Sample 4 |
| **Bilirubin** | 0 | 164.38 | 5064.22 | 20.23 | 1104.25 |
|  | 818 μmol/L | 167.55 | 4954.33 | 19.29 | 1074.52 |
|  | Bias | 1.93% | -2.17% | -4.65% | -2.69% |
| **Hemoglobin** | 0 | 164.2 | 5127.75 | 20.93 | 1081.31 |
|  | 18 g/L | 157.33 | 5019.9 | 20.7 | 1067.55 |
|  | Bias | -4.18% | -2.10% | -1.10% | -1.27% |
| **Triglyceride** | 0 | 168.53 | 5172.7 | 21.25 | 1079.39 |
|  | 21.54 mmol/L | 164.62 | 5080.92 | 21.9 | 1104.71 |
|  | Bias | -2.32% | -1.77% | 3.06% | 2.35% |
| **Rheumatoid factor** | 0 | 118.27 | 3605.68 | 15.38 | 724.17 |
|  | 1500 IU/mL | 122.18 | 3438.13 | 14.78 | 754.67 |
|  | Bias | 3.31% | -4.65% | -3.90% | 4.21% |
| **Antinuclear antibody** | 0 | 178.04 | 5285.83 | 21.44 | 1152.01 |
|  | 500 ng/mL | 180.18 | 5340.41 | 22.4 | 1201.98 |
|  | Bias | 1.20% | 1.03% | 4.48% | 4.34% |
| **Human anti-mouse antibody** | 0 | 177.59 | 5172.98 | 21.2 | 1153.01 |
|  | 500 ng/mL | 184.33 | 5377.82 | 21.35 | 1152.76 |
|  | Bias | 3.80% | 3.96% | 0.71% | -0.02% |

**Supplementary Table 9** Judgment of abnormality in level of sFlt-1

| **Test sFlt-1 kit** | **Roche sFlt-1 kit** | | |
| --- | --- | --- | --- |
|  | Normal | Abnormal | Total |
| Normal | 117 | 0 | 117 |
| Abnormal | 0 | 56 | 56 |
| Total | 117 | 56 | 173 |

**Supplementary Table 10** Judgment of abnormality in level of PlGF

| **Test PlGF kit** | **Roche PlGF kit** | | |
| --- | --- | --- | --- |
|  | Normal | Abnormal | Total |
| Normal | 116 | 0 | 116 |
| Abnormal | 1 | 56 | 57 |
| Total | 117 | 56 | 173 |


**Supplementary Table 11 Comparison of different detection metrics in the discovery set**

| **Total** | **1-Specificity** | **Specificity** | **Sensitivity** |
| --- | --- | --- | --- |
| sFlt-1 (Test kit) | 0.167 | 0.833 | 0.524 |
| PlGF (Test kit) | 0.167 | 0.833 | 0.500 |
| sFlt-1/PlGF  (Test kit) | 0.167 | 0.833 | 0.762 |
| sFlt-1 (Roche kit) | 0.167 | 0.833 | 0.500 |
| PlGF (Roche kit) | 0.167 | 0.833 | 0.500 |
| sFlt-1/PlGF  (Roche kit) | 0.167 | 0.833 | 0.714 |

**Supplementary Table 12 Comparison of different detection metrics in the validation set**

| **Total** | **1-Specificity** | **Specificity** | **Sensitivity** |
| --- | --- | --- | --- |
| sFlt-1 (Test kit) | 0.167 | 0.833 | 0.623 |
| PlGF (Test kit) | 0.167 | 0.833 | 0.451 |
| sFlt-1/PlGF  (Test kit) | 0.167 | 0.833 | 0.679 |
| sFlt-1 (Roche kit) | 0.167 | 0.833 | 0.623 |
| PlGF (Roche kit) | 0.167 | 0.833 | 0.611 |
| sFlt-1/PlGF  (Roche kit) | 0.167 | 0.833 | 0.774 |
